# Septic Cavernous Sinus Thrombosis in a Young Male Presenting With Pneumothorax Secondary to Septic Pulmonary Emboli: A Case Report

**DOI:** 10.7759/cureus.35636

**Published:** 2023-03-01

**Authors:** Roopeessh Vempati, Kavya Balusu, Aishwar Dixit, Sri Anugna Miriyala, Rishika Masna, K. ShashiVardhan Reddy, Sanjana Devaragudi, Afrasayab Khan, Sweta Sahu, Vishal Venugopal

**Affiliations:** 1 Internal Medicine, Gandhi Medical College and Hospital, Hyderabad, IND; 2 Internal Medicine, Gandhi Hospital, Hyderabad, IND; 3 Internal Medicine, Baba Raghav Das Medical College, Gorakhpur, IND; 4 Internal Medicine, Kamineni Institute of Medical Sciences, Nalgonda, IND; 5 Internal Medicine, Kamineni Institute of Medical Sciences, Hyderabad, IND; 6 Medical School, Osmania Medical College, Hyderabad, IND; 7 General Medicine, Apollo Institute of Medical Sciences and Research, Hyderabad, IND; 8 Internal Medicine, Directorate of Health Services, Jammu and Kashmir, Ramban, IND; 9 Internal Medicine, Government Medical College, Srinagar, Srinagar, IND; 10 Surgery, Jagadguru Jayadeva Murugarajendra Medical College, Davanagere, IND; 11 Internal Medicine, Bhaarath Medical College and Hospital, Chennai, IND

**Keywords:** septic cavernous sinus thrombosis, pneumothorax ptx, pneumothorax, septic pulmonary emboli, septic pulmonary embolism

## Abstract

Septic pulmonary embolism (SPE) is a rare complication that happens when infected thrombi from the original site of infection break off and travel to the pulmonary blood vessels, causing an infarction or an abscess. Cases were reported on SPE, with tricuspid or pulmonary valve endocarditis being the most common primary site, especially in intravenous drug abusers. There are, however, very few reports of SPE brought on by septic cavernous sinus thrombosis (CST). Here, we describe the case of an 18-year-old male who had a pustule on his left eyelid, after which he developed fever, spontaneous swelling of his left eye, followed by his right eye, along with bilateral proptosis and diplopia, and new-onset dyspnea. Auscultation revealed decreased breath sounds in the left lung fields. Magnetic resonance imaging (MRI) revealed cavernous sinus thrombosis. Blood cultures isolated *Staphylococcus aureus* species. High-resolution computed tomography (HRCT) revealed a left-sided pneumothorax with minimal pleural effusion and multiple nodules scattered among both lungs, suggesting septic pulmonary emboli. We report this case to convey how a minor lesion, that is, an eyelid pustule (stye), can get complicated and set off a spiral of events that takes an unexpected tangent, challenging physicians and necessitating a rigorous approach.

## Introduction

The paired cavernous sinuses on each side of the sella tursica are formed by the dural layers and contain venous blood draining from the cranial cavity via the sphenoparietal sinuses, cerebral veins, and superior ophthalmic veins. Through emissary veins, the cavernous sinuses also connect to the deep facial veins, the inferior ophthalmic veins, and the pterygoid plexus [1]. The cavernous sinuses then drain through the petrosal sinuses into the sigmoid sinuses and internal jugular veins [2].

Cavernous sinus thrombosis (CST) is a rare condition with a high death rate. It is usually caused by a septic aetiology and less often by an aseptic aetiology like trauma, surgery, or pregnancy, especially when it happens with a thrombophilic condition [1]. A sinus infection causes blood clotting, which causes CST [3]. Bacteria cause the majority of septic cases, with *Staphylococcus aureus *being the most commonly implicated agent. *Streptococcus *is the second-most common cause. Cavernous sinus thrombosis can also be caused by gram-negative rods, anaerobes, and fungal agents [4]. Septic cavernous venous sinus thrombosis generally presents with fever, headache, proptosis, ophthalmoplegia, and variable visual impairment. If left untreated, the infection is almost uniformly fatal. The widespread use of effective antibiotic therapy has improved the prognosis, but the disease is still associated with significant mortality and morbidity [5]. Ludlow did a study in 1852 to find out how the "danger triangle of the face," which is the area from the corners of the mouth to the bridge of the nose and includes the nose and maxilla, is related to the risk of cavernous sinus thrombosis [6].

Due to the lack of valves in the dural venous sinuses, blood can flow in either direction, which allows infectious agents to reach different parts of the brain and lead to complications such as meningitis, dural empyema, or brain abscess. This characteristic is of significance because pathogens can travel from the face into the brain via the cavernous sinuses [2]. Most unilateral infections of the cavernous sinuses end up being bilateral due to communication between them via the intercavernous sinuses. Infection can also spread via the jugular vein to the pulmonary vasculature, resulting in septic emboli or abscesses, pneumonia, or empyema [1]. In addition, the oculomotor nerve, the trochlear nerve, the ophthalmic and maxillary branches of the trigeminal nerve, the abducens nerve, and the internal carotid artery are susceptible to inflammatory damage as they are adjacent to the cavernous sinuses, which can lead to various complications [7].

## Case presentation

**Figure 1:**
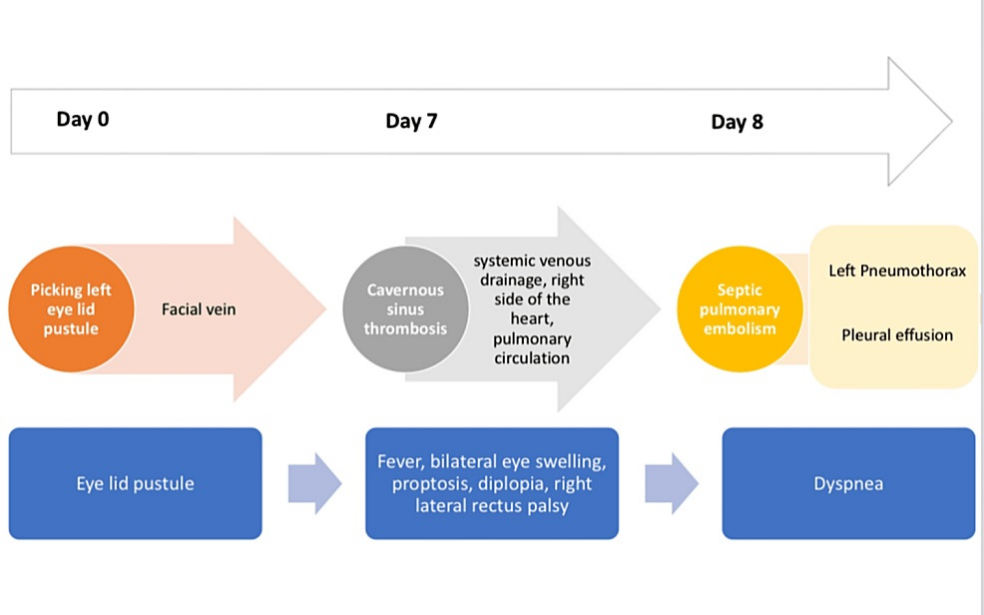
Progress of the disease at the time of presentation

An 18-year-old male without any comorbidities and no known addictions presented to the emergency department with complaints of fever since 6 days, bilateral swelling and protrusion of the eyes since 3 days, and shortness of breath since 2 days. A pustular lesion over his left eyelid (stye) appeared a week ago, which he picked, and the next day he developed a fever (Figure 1).

The fever began as low grade, then progressed to high grade, was continuous, was accompanied by chills, and was not relieved by antipyretic medication. Three days later, the patient noticed a spontaneous and sudden swelling of the left eyelid. The swelling was painful, red, and associated with the protrusion of the eye. The next day, the patient developed a similar swelling in the right eye. He then developed double vision. There have been no reports of blurred vision or headaches, nor of coughing or hemoptysis. On examination, the patient was conscious, coherent, and cooperative. The vital signs of the patient on admission were a blood pressure of 110/70 mmHg, 78 beats per minute for the heart, 18 cycles per minute for the respiratory rate, a saturation of 98% for oxygen in room air, and he was febrile. There were no signs of pallor, icterus, cyanosis, edema, or lymphadenopathy. There was a deviated nasal septum and congestion of the nasal mucosa on the left side. The paranasal sinuses were not tender. Auscultation revealed normal heart sounds, and bilateral air entry was present in both lungs with decreased breath sounds in the left infrascapular and infra-axillary areas and normal vesicular breath sounds in the right lung. A central nervous system examination revealed right lateral rectus palsy and left lower motor neuron facial nerve palsy, and the plantar reflex was downward on both sides. Ophthalmological examination showed bilateral proptosis with an inability to close the eyelids, and fundoscopy was within normal limits. In the peripheral blood smear, they saw normocytic hypochromic, neutrophil leukocytosis with a slight shift to the left, and thrombocytosis.

The investigations carried out are detailed in Table 1.

**Table 1:** General investigations of the patient AST: Aspartate Aminotransferase; ALT: Alanine Transaminase; ALP: Alkaline Phosphatase; ESR: Erythrocyte Sedimentation Rate

Parameter	Patient’s report	Reference range	Comment
Total Bilirubin (mg/dL)	0.9	0.1-1.0	Normal
Direct Bilirubin (mg/dL)	0.2	0.0-0.3	Normal
AST (U/L)	22.5	12-38	Normal
ALT (U/L)	44.4	7- 56	Normal
ALP (U/L)	101.9	44-147	Normal
Total protein (g/dl)	5.88	6 - 7.8	Low
Albumin (g/dl)	2.17	3.5 - 5.5	Low
Globulin (g/dl)	3.71	2.3 - 3.5	High
Blood Urea (mg/dl)	23	19 - 44	Normal
Blood urea nitrogen	7.7	7-18	Normal
Serum creatinine (mg/dl)	0.8	0.6-1.2	Normal
Serum sodium (meq cs/L)	136	135 - 145	Normal
Serum potassium (meq/L)	4	3.5 - 5	Normal
Serum chloride (meq/L)	101	95 - 105	Normal
Random Blood Sugar (mg/dl)	127.1	100 - 140	Normal
ESR (mm/hour)	140	0 - 22	High

Day-wise blood profile was tested (Table 2).

**Table 2:** Blood investigations of four consecutive days WBC: White Blood Cells

	Reference Range	Day 1	Day 2	Day 4	Day 6
Hemoglobin (g/dl)	13.2-16.6	13	11.1	12.8	12.6
WBC (cells/mm3)	4,500-11,000	16,800	28,300	23,460	18,410
Platelets (cells/mm3)	1,50,000-4,00,000	3,46,000	3,54,000	3,67,000	4,75,000
C-Reactive protein (mg/L)	3-10	73	-	192	-

The fever profile showed all the parameters being negative (Table 3).

**Table 3:** Fever profile of the patient ELISA: enzyme-linked immunosorbent assay; NS1: nonstructural protein 1 of dengue

Widal test (tube agglutination)	Negative
Plasmodium falciparum and vivax (Ig-M)	Negative
Chikungunya (Ig-M ELISA)	Negative
Dengue (NS-1 and Ig-M)	Negative

Normocytic hypochromic, neutrophil leukocytosis with a slight leftward shift and thrombocytosis were observed in the peripheral blood smear. An MRI of the brain showed signs of cavernous sinus thrombosis, and the left cerebellar lobe probably had cerebritis or a venous infarct. The high-resolution CT (HRCT) chest revealed a left-sided pneumothorax with minimal effusion, a thick-walled cavitary lesion in the left upper lobe of the lung in the subpleural region, and diffuse scattered nodules in both lung fields, all of which point to septic pulmonary emboli. A CT scan of the paranasal sinus revealed deviation of the bony septum to the right side with a bony spur, left inferior turbinate hypertrophy, and left concha bullosa, along with mucosal thickening noted in bilateral sphenoid sinuses and polypoidal mucosal thickening noted in the right maxillary sinus. COVID-19 rapid antigen test done at admission was negative. 2D echocardiography was within normal ranges. 

Management

The patient was admitted for acute medical care and was put on high-flow oxygen inhalation for pneumothorax resolution. Foley catheterization was done, a urine sample was collected in a sterile container for culture, and the patient was advised to have strict bed rest. Peripheral venous cannulation was done, and venous blood samples were drawn for routines and also for culture and sensitivity before initiation of treatment. At a rate of 75 ml/hour, fluids were given through an IV that included one pint of normal saline, one pint of dextrose-normal saline, and two pints of normal saline. Serial chest X-ray monitoring was ordered, and vitals were monitored strictly.

The patient was then given injections of low-molecular-weight heparin (40 mg subcutaneously twice daily (BID)) and aspirin (150 mg per oral (PO) OD (once daily), decreasing to 75 mg PO OD on the second day). Both of these treatments were kept up until the last day of hospitalization.

Additionally, the patient was put on empirical therapy with injections of meropenem 1 g TID (thrice a day) and injections of clarithromycin 500 mg BID intravenously, which were changed to injections of linezolid 600 mg and ceftriaxone 1 g, given by intravenous route, after the blood cultures had grown methicillin-sensitive *Staphylococcus aureus* (MSSA). MSSA was isolated from blood and is resistant to erythromycin but susceptible to co-trimoxazole, cefotaxime, amoxiclav, and linezolid. No bacterial growth was detected by urine culture.

Seizure prophylaxis was given with injections of levetiracetam 500 mg BID.

Also, the patient was given an intravenous infusion of 1 g of acetaminophen every 12 hours (BID) until the fever went away. After that, he was given 500 mg of acetaminophen tablets TID. Injections of pantoprazole (40 mg) and ondansetron (4 mg) were also given intravenously.

After the patient had been stabilized, ultrasound-guided aspiration was done for pleural effusion in the left lung. When a USG chest was performed to aspirate pleural fluid, it revealed a mild pleural effusion in the left lung that appeared organised and had multiple septations, suggesting an infectious cause. It also showed that the bottom parts of the right lung had grown together and that the left dome of the diaphragm moved less.

After 10 days in the hospital, the patient no longer had a fever, and the swelling in both eyes went down. The patient was sent home with linezolid 600 mg BID tablets and cefixime 200 mg BID tablets to be taken by mouth for 6 days. At the initial follow-up, the patient remained healthy, and at a follow-up 6 months later, he still had a right lateral rectus palsy.

## Discussion

The patient had a pustular lesion (stye) on the left eyelid that had been present for one week and was evidently the cause of the septic CST due to the temporality of its clinical features. Due to the interconnections between the bilateral cavernous sinuses, he had involvement in both eyes. The right lateral rectus palsy was caused by the involvement of the right abducens nerve due to its proximity to the cavernous sinus. The best way to diagnose CST is through clinical evidence, which is then supported by radiographic tests. In this instance, an MRI was performed to aid in an early diagnosis. It is best to perform an MRI as soon as possible with gadolinium enhancement when septic CST is suspected [7].

An HRCT of the chest showed that a septic pulmonary emboli led to a secondary spontaneous pneumothorax with minimal effusion. These pulmonary emboli may have been a hematogenous extension from the CST through the right side of the heart to the lungs. Throughout his hospital stay, the patient was on anticoagulant and antiplatelet therapy. Anticoagulation with unfractionated heparin or low-molecular-weight heparin has only been shown to reduce morbidity, ophthalmoplegia, blindness, stroke, and seizures but not overall mortality [8]. The benefit would be to stop the progression of thrombosis, stop the spread of clots, and perhaps allow antibiotics to penetrate; the risk, on the other hand, would be systemic or intracranial bleeding or even the spread of septic emboli.

Facial infections, acute sinusitis, and periorbital infections are the main risk factors. Cavernous sinus thrombosis can be brought on by a variety of infectious agents, most of which are bacterial. Two-thirds of cases may be attributable to *Staphylococcus aureus*, and methicillin resistance should be taken into account. On the second day of admission, the blood culture revealed a *Staphylococcus aureus *infection. An empirical antibiotic was first administered to the patient, and then culture-specific antibiotics were administered. This early antibiotic administration was extremely beneficial in containing the infection. 

Gram-positive cocci, like *Staphylococcus aureus *and streptococcal species, are the most frequent causes. Empiric therapy involves administering a beta-lactamase-resistant penicillin or cephalosporin early on to treat these organisms [7].

The most frequent causes of death from CST are sepsis and CNS infections, which had a fatality rate of almost 100% before the development of potent antibiotic therapies. Because of strict management, the death rate has dropped below 30%, but morbidity is still high and full recovery is still rare. The late diagnosis and use of antibiotics could be to blame for these high death and illness rates [9].

A comparison of various outcomes from similar cases along with our case was done for better understanding. The outcomes of studies conducted by Barranco-Trabi J, et al [10], Allegrini D, et al [11], and Schear MJ, et [12] were considered and compared with our case. The comparison is given in Table 4.

**Table 4:** Comparison of various other cases with this case

	Hoshino C, et al 2007 {7]	Kotagiri R, et al 2020 [8]	Barranco-Trabi J, et al 2022 [10]	Allegrini D, et al 2017 [11]	Schear MJ, et al 2012 [12]	This case
Age/Gender	56/female	35/female	21/male	46/female	38/male	18/male
Source of infection	Upper respiratory tract infection	Unknown	Picking acne on nose	Odontogenic origin	Nasal abscess	Picking of eyelid pustule
Clinical features	Fever, sore throat, right-sided headache, right eyelid swelling.	Neck stiffness is associated with sharp neck pain, fever, headaches, diplopia, and left-sided ptosis.	Facial swelling, painful rash on the nose, nasal swelling and nasal discharge, pain with eye movements, headache, nausea, anorexia, photophobia, neck stiffness, shortness of breath, and subjective fevers.	Facial pain, eyelid swelling, and proptosis with ophthalmoplegia in the left eye.	Bilateral ptosis, facial puffiness, motility dysfunction	Fever, eye swelling, diplopia, and shortness of breath
Complications	External ophthalmoplegia, trigeminal neuralgia, the narrowing of the right internal carotid artery at the intercavernous portion, subarachnoid abscess, and bilateral septic pulmonary emboli.	Right lateral palsy, left partial oculomotor nerve palsy, left acute non-hemorrhagic posterior cerebral artery territory infarct, encephalopathy, and bilateral septic pulmonary emboli.	bilateral septic pulmonary emboli.	Massive bilateral pulmonary artery thrombosis and an infarct in the superior segment of the basal lobe of the right lung.	Left temporalis muscle abscess; central retinal artery occlusion of the left eye; bilateral septic pulmonary emboli.	Right lateral rectus palsy, septic pulmonary emboli with left pneumothorax and pleural effusion
Therapeutic anticoagulation/antiplatelet regimen	200 mg/day of Cilostazol	Unfractionated heparin received for all days	Heparin drip without bolus dose followed by enoxaparin for a 7 day course	Heparin for 10 days	Unfractionated heparin given for 13 days, later bridged to warfarin	Low-molecular weight heparin and aspirin throughout the stay
Empirical antibiotic therapy	Sulbactam/ampicillin	Vancomycin and ceftriaxone	Vancomycin and piperacillin/tazobactam.	N/A	Vancomycin	Meropenem, clarithromycin
Organism isolated from blood cultures	Streptococcus constellatus	Methicillin resistant Staphylococcus aureus (MRSA)	Methicilin sensitive Staphylococcus aureus (MSSA)	Streptococcus constellatus	Methicilin sensitive Staphylococcus aureus (MSSA)	Methicilin sensitive Staphylococcus aureus (MSSA)
Antibiotic therapy after culture report	Sulbactam/ampicillin	Ceftaroline and vancomycin. Ceftaroline was replaced with daptomycin as the patient developed a drug rash. Vancomycin was replaced with linezolid to improve central nervous system (CNS) penetration.	Oxacillin and gentamicin. Oxacillin was switched to cefazolin owing to mild hypertransaminasemia.	Piperacillin/ tazobactam	Vancomycin	Ceftriaxone, linezolid
Other management strategies	N/A	Intubation.	N/A	N/A	Lateral canthotomy and cantholysis of the left eye; dexamethasone for orbital inflammation.	High-flow oxygen inhalation for pneumothorax resolution.
Outcome	recovery; right lateral gaze palsy persisted.	Death	Recovery.	Recovery.	recovery; bilateral lateral rectus palsy persisted.	Recovery; right lateral rectus palsy persisted.

## Conclusions

Septic pulmonary embolism (SPE) is an unexpected sequela, and on top of that, SPE from a CST is unusual. Identifying the primary site of infection along with isolating the culprit microbe helped us in understanding the case and converging the antimicrobial therapy to the specific microbe. In addition, optimal duration of specific antibiotic therapy and an anticoagulation/antiplatelet regimen aided in the resolution of infective and thrombotic clinical features. After clinical resolution, our patient was released with oral antibiotics and advised to follow up.
